# Association of Parental Factors and Insulin-like Growth Factor 2 Polymorphism with Intrauterine Growth Restriction

**DOI:** 10.3390/children9050630

**Published:** 2022-04-28

**Authors:** Monica G. Hăşmăşanu, Sorana D. Bolboacă, Lucia Maria Procopciuc, Melinda Matyas, Ligia Blaga, Daniel Mureșan, Gabriela C. Zaharie

**Affiliations:** 1 Department of Neonatology, Iuliu Hațieganu University of Medicine and Pharmacy, 400006 Cluj-Napoca, Romania; popa.monica@elearn.umfcluj.ro (M.G.H.); melinda.matyas@umfcluj.ro (M.M.); ligia.blaga@umfcluj.ro (L.B.); gzaharie@umfcluj.ro (G.C.Z.); 2 Department of Medical Informatics and Biostatistics, Iuliu Hațieganu University of Medicine and Pharmacy, 400349 Cluj-Napoca, Romania; 3 Department of Medical Biochemistry, Iuliu Hațieganu University of Medicine and Pharmacy, 400349 Cluj-Napoca, Romania; lprocopciuc@umfcluj.ro; 4 Department of Obstetrics and Gynecology, Iuliu Hațieganu University of Medicine and Pharmacy, 400006 Cluj-Napoca, Romania; daniel.muresan@umfcluj.ro

**Keywords:** insulin-like growth factor 2 (IGF2) polymorphism, intrauterine growth restriction (IUGR), parental factors

## Abstract

Polymorphism of insulin-like growth factor 2 (IGF2) is known to play a role in cell development. Only the paternal IGF2 copy is active, while the copy inherited from the mother is inactive. This study aimed to explore whether maternal and paternal factors influence IGF2 polymorphism in newborns with intrauterine growth restriction (IUGR) compared to appropriate for gestational age (AGA). A cross-sectional exploratory study was conducted from June 2014 to November 2015 at the Neonatology, Gynecology 1 Clinic, Cluj-Napoca, Romania. The ApaI IGF2 genotypes and allele frequencies were similar in the IUGR and AGA groups (*p*-value > 0.10). The IUGR babies with a protective IGF2 genetic profile had significantly younger parents (a difference in the median age of 8 years for mothers and 9 years for fathers; *p*-value < 0.009). The IUGR babies had parents with lower birth weights than AGA babies (mothers’ medians: 2800 g vs. 3100 g; fathers’ medians: 3000 g vs. 3400 g; *p*-value < 0.02). In univariable regression analysis, the mother’s and father’s birth weight proved to be associated with IUGR. The father’s birth weight proved to be the only factor significantly associated with IUGR, independent of the mother’s birth weight or the presence of a protective IGF2 genetic profile (odd ratio = 0.998 [0.996 to 1.000], *p*-value = 0.032).

## 1. Introduction

Personal experiences determine the individual’s health during existence. Fetal programming occurs during embryonic and fetal development, a critical period in which tissues and organs are created [1]. The critical period coincides with the time of rapid differentiation of cells, and factors that act during this period permanently affect the structure and subsequent function of the body. Environmental factors, in particular, nutrition during pregnancy and early life, may influence the risk of chronic diseases in later life [2]. However, the mechanism of fetal disease programming in adults remains a hypothesis. Insufficient understanding of the mechanism exists, and no method to determine the predisposition to disease in adulthood is known. Therefore, it is necessary to study the mechanisms at the cellular and molecular level.

The etiology of intrauterine growth restriction (IUGR) is multifactorial and it is based on maternal, fetal, placental, and genetic mechanisms. Maternal risk factors for IUGR include smoking during pregnancy, low pre-pregnancy weight, and low weight gain. Risk factors more strongly associated with preterm IUGR include chronic hypertension and advanced maternal age. Furthermore, low pre-pregnancy body mass index has been associated with an increased risk of term IUGR. Any specific ischemic placental condition in a prior pregnancy is also a risk factor for that same ischemic placental disease in a subsequent pregnancy [3]. Paternal age significantly affects birth weight, with advanced paternal age increasing the risk of small for gestational age among preterm infants and decreasing the risk among infants born at term [4]. Expression of the IGF2 paternal gene is inherited in an autosomal-dominant mode. Still, according to the imprinting status of the genes, the growth disturbance phenotypes only occur in the case of paternal (IGF2, 11p15.5) or maternal (CDKN1C) inheritance. The pathogenic variants of the paternally expressed IGF2 gene are associated with growth retardation [5].

The human species inherits a copy of most genes from the mother and a copy from the father. Both copies are usually active or “activated” at the cellular level [6]. However, the activity of the insulin-like growth factor 2 (IGF2) gene depends on which of the parents it was inherited from. Only the paternal copy is active for IGF2, while the copy inherited from the mother is inactive. This specific parental difference in gene activation is known as genomic imprinting [6]. Circulating IGF2 is mainly synthesized by the liver, where, unlike other tissues, IGF2 is expressed biallelically.

In mice, interruption of the paternal IGF2 allele causes a severe delay in prenatal growth [7] and postnatal growth [8], an effect that has not been demonstrated in the human population. In human development, a key factor is the insulin-like system IGF2 and insulin-like growth factor 2 receptor (IGF2R). Low levels of circulating IGF-2 (IGF-2 < 400 ng/mL) are associated with an increased risk of weight gain and obesity [9].

The ApaI polymorphism of insulin-like growth factor 2 (IGF2) had been reported to be associated with body mass index, fat mass, grip strength [10] and obesity [11] in adult population. Few studies analyze the link between development and growth in the neonatal population and the ApaI IGF2 polymorphism. The IGF2 and ApaI polymorphism proved no association neither in low birth weight nor in average birth weight [12]. The IGF2 ApaI polymorphism in partners of recurrent spontaneous abortion (RSA) women could affect the IGF2 level of expression in the placenta and embryo and represent a risk factor for RSA susceptibility [13].

Our study aimed to explore whether maternal and paternal factors influence IGF2 polymorphism in intrauterine growth restriction (IUGR) newborns compared to appropriate for gestational age (AGA) newborns.

## 2. Materials and Methods

### 2.1. Study Design and Settings

A cross-sectional exploratory study was conducted to evaluate several parental (maternal and paternal) factors’ on the ApaI IGF2 polymorphism in newborns with IUGR and AGA. Maternal and paternal anthropometric data, pre-pregnancy and mother’s pregnancy-related pathology, mother weight gain during pregnancy, consumption of toxins (alcohol, number of cigarettes), number of pregnancies, and birth weight of parents were collected. Parents’ body mass index was calculated, and subjects were classified as overweight or obese by body mass index (BMI) [14].

Newborns with IUGR diagnosed by fetal ultrasound evaluation and subsequently hospitalized in the Department of Neonatology Gynecology 1 Clinic (Cluj-Napoca, Romania), level 3 maternity, for 1.5 years (June 2014–November 2015) were included in the study. The AGA group was newborns of the same gestational age as the IUGR group. Newborns with any chromosomopathy or malformation were excluded. 

The main characteristics of the evaluated groups were previously reported in the scientific literature [15]. Sixty-one newborns were evaluated, 40 in the IUGR group and 21 in the AGA group. No significant deviation from the Hardy–Weinberg equilibrium evaluated following the Elston and Forthofer method [16] was observed in the studied cohort (whole cohort and individual groups). The ApaI IGF2 genotypes and allele frequencies were similar in the IUGR and AGA groups (*p*-value > 0.10 [15]). The IUGR mothers’ characteristics proved not significantly different from the AGA mothers’ characteristics: age (*p*-value = 0.122), mothers’ pre-pregnancy BMI (*p*-value > 0.10), ethnicity (*p*-value > 0.2), and mother’s smoking status (*p*-value > 0.4) [15]. The newborns in the IUGR group had significantly higher IGF2 values (77.60 pg/mL (48.00–127.00) vs. 59.30 pg/mL (47.30–167.45) in the AGA group; *p*-value = 0.049) [15].

### 2.2. Statistical Analysis

Statistical analysis was made with IBM SPSS Statistics for Windows software (Version 28.0.0 trial version, IBM Corp., Armonk, NY, USA) at a significance level of 5%, so the *p*-values less than 0.05 were considered statistically significant. 

Measured data were summarized as mean ± standard deviation whenever data proved to follow the theoretical normal distribution (Shapiro–Wilk test); otherwise, median and IQR (interquartile range defined as (Q1–Q3), where Q1 is the first quartile and Q3 is the third quartile) were reported. The chi-squared or Fisher exact test, or Z-test for the proportion were used to compare IUGR with AGA whenever qualitative variables were tested. The Student t–test for normally distributed quantitative data or otherwise the Mann–Whitney test was used for comparisons between IUGR and AGA groups. 

All parents and babies (IGF2 values [15] and ApaI IGF2 genetic profile as protective–AA genotype and non-protective) characteristics with *p*-values ≤ 0.10 in an univariable exploratory regression analysis were considered as potential factors for IUGR and entered into multivariable logistic regression analysis. The ApaI IGF2 genotype (protective vs. non-protective) was included in the multivariable logistic regression analysis as a possible confounder regardless of the univariable logistic regression result. The effect of factors on IUGR was expressed as an odd ratio, inclusive of the associated 95% confidence bounds and significance.

## 3. Results

The birth weight of both mother and father of a child with IUGR proved to be significantly lower than the parents of children with AGA (Table 1). A significantly higher percentage of fathers of an AGA child were overweight compared to the fathers of IUGR children. In comparison, a significantly higher percentage of the fathers of IUGR children had grade I obesity than the fathers of AGA children (Table 1).

No mother declared alcohol consumption either in the IUGR or in AGA group. Most mothers declared no medical history (23/40 in the IUGR group and 18/21 in the AGA group, *p*-value = 0.0258). Two mothers in the IUGR group had blood hypertension, and one in the AGA group had diabetes. Fifteen mothers in the IUGR group (37.5%) and two in the AGA group (9.5%) had other diseases (such as neurofibromatosis, nodular goiter, hyperprolactinemia, hepatitis B, rheumatoid arthritis, peritonitis operated, myeloproliferative syndrome, thrombocytosis, spherocytosis, splenomegaly, myalgia, operated omphalocele, operated tuberculosis, gastritis, hypothyroidism, Hashimoto’s thyroiditis, thrombophilia, right lung pleurisy). As a result, the mothers with an IUGR baby reported a specific disease significantly more frequently than the mothers of an AGA baby (*p*-value = 0.0205).

Like mothers, most fathers had no medical history (37/40 in the IUGR group and 21/21 in the AGA group). One father of a child with IUGR reported hypertension, one reported diabetes, and another hepatitis B.

Ten children, five with IUGR (12.5%) and five with AGA (23.8%), had an IGF2 protective genetic profile (AA genotype). No significant association was identified between non-protective genetic profile and IUGR/AGA (Fisher exact test: *p*-value = 0.2170). In the IUGR group, the parents (mother and father) of children with a protective genetic profile (AA genotype) were significantly younger as compared to the parents of children with non-protective profiles (AG or GG) (Table 2).

The univariable and multivariable logistic regression analysis showed that non-protective IGF2 genetic profile (AG or GG) is not significantly associated with IUGR. The fathers’ birth weight is associated with IUGR independent of the IGF2 genetic profile (Table 3).

## 4. Discussion

Our results showed that children with intrauterine growth restriction had parents (mother and father) with lower birth weight than children appropriate for gestational age. Still, only the father’s birth weight remained significantly associated with IUGR in multivariable regression analysis, independent of the ApaI IGF2 genotype.

Alcohol and drug abuse compromise maternal nutritional status. Thus, the supply of essential nutrients is not available for the fetus and can lead to fetal abnormalities like IUGR or Fetal Alcohol Spectrum Disorder (FASD) [17]. In our study, only fathers declared alcohol consumption (Table 1) but without differences between IUGR and AGA groups (*p*-value > 0.5). 

Another critical factor in developing IUGR is the maternal pathology during pregnancy, with a particular contribution of preeclampsia (PE). A subset of women develop early-onset PE and are diagnosed with IUGR in special early preeclampsia [18], suggesting an overlap in the etiology underlying these complications [19]. The mother’s medical history was analyzed, but most mothers (>55%) did not declare any positive medical history in our study. Hypertension was present in two mothers with IUGR babies, but other diseases were more frequently reported in this group than in the AGA group (*p*-value < 0.05). Maternal pathology and preeclampsia can induce disorders in the development of the placenta, and thus there are changes in placental membrane transporters and in the expression of these transporters. A systematic shift in nutrient transporter expression was detected, a factor that could lead to IUGR [20].

Maternal age and ethnicity, and socio-economic and marital status can be associated with child IUGR, and these factors may explain how maternal cultural background and environment influence small for gestational age patterns in a specific population [21]. In our study, we observed no differences of mother’s and father’s age and ethnicity among the evaluated groups (Table 1).

Analyzing the influence of genetic factors, it is known that several imprinted genes are associated with body weight dysregulation. In humans, genetic disorders affecting imprinted genes lead to obesity [22]. Obesity, defined as an increase in white adipose tissue (WAT) [23] mass, arises from a complex interaction between genetic and environmental factors. Over the last years, the obesity rate has increased rapidly in industrial countries. It has led to an increase in obesity-associated metabolic diseases, such as type 2 diabetes and cardiovascular disease. In our study, overweight in the father was more common in the AGA group than in IUGR group (Table 1). Therefore, we must gain a greater understanding of the molecular and genetic mechanisms governing the development of obesity. The paternal transmission of obesity in mice was correlated with the expression of the imprinted genes for IGF2, which might contribute to the symptoms associated with obesity [22].

Our results reported no significant association between IGF2 polymorphism and IUGR, similar to results reported by de Mascena Diniz Maia et al. [12]. We found only in the IUGR group an association between the protective profile (AA genotype) and the parents’ age (Table 2). Meaning the parents (mother and father) of babies with protective genetic profiles (AA genotype) are significantly younger as compared to the parents of babies with non-protective profiles (AG or GG). The literature has not described this association between the genetic profile and the parents’ age. Our result must be interpreted with appropriate attention because the number of IUGR babies with AA genotype is very small and thus, this observed difference needs to be tested with a proper sample size. Whether the father’s age could be attributed to a protective role in the evolution of newborns with IUGR need to be further investigated. It is known that preconception maternal obesity is a risk factor for IUGR [24]. The evidence suggests that maternal and paternal periconceptional nutrition indicates the offspring metabolic syndrome risk in later life through epigenetic imprinting [25]. Our study could not demonstrate differences between maternal [15] or paternal BMI that influence the risk of IUGR (Table 1). However, in the NEST cohort (Newborn Epigenetics Study), genetic analysis of IGF2 expression showed that paternal obesity is associated with IGF2 hypomethylation in newborns [26]. 

Our study demonstrated lower parental birth weight among IUGR babies than AGA babies (Table 1). In univariable analysis, parentals’ birth weight proved to be significantly associated with IUGR, but no significant association of parental’s age, birth weight, or IGF2 genetic profile was demonstrated (Table 3). In multivariable analysis, fathers’ birth weight remained statistically associated with IUGR (Table 3), with younger fathers having a protective effect. Although fathers’ birth weight demonstrated statistical significance (Table 3), this association could emerge in either direction when the number of evaluated subjects increases since the upper bound of the 95% confidence interval is exactly 1, with a value equal to 1 indicating the same risk in the IUGR and AGA groups. Advanced paternal age increases the risk of small for gestational age among preterm infants and decreases the risk among infants born at term [12]. Qian et al. [27] reported on a Taiwan sample that children born by IUGR mothers are more likely to have IUGR, but children born from an IUGR father are not. They suggest that maternal health is pertinent and that socio-economic intervention may not yield the desired outcomes within a short period [27].

The main limitation of our study is given by the nature of the design, namely, an exploratory study, and thus with a small sample size. The association of maternal and paternal factors with the genetic polymorphism ApaI IGF2 could be valid, but the reported results require validation in larger samples. The relationship between parental birth weight and IGF2 polymorphism in newborns with IUGR and AGA also needs to be validated in larger samples. Thus, our results indicate what we observed in the investigated cohort and generalizing from them is inappropriate. 

## 5. Conclusions

Our results showed that children with intrauterine growth restriction (IUGR) had parents (mother and father) with lower birth weight than children appropriate for gestational age (AGA). Moreover, AGA (appropriate for gestational age) babies had an IGF2 (insulin-like growth factor 2) protective genetic profile (AA genotype) more frequently than IUGR (intrauterine growth restriction) babies, although the threshold of significance was not reached. Babies with IGF2 protective genetic profiles in the IUGR group had younger parents than those without IGF2 protective genetic profiles. Still, this result could be observed by chance since the protective profile was observed only in five babies and thus needs validation with larger samples. Only the father’s birth weight remained significantly associated with IUGR in the multivariable logistic regression analysis, but the upper bound of the 95% confidence interval indicates a low relevance. Our results indicate a possible independent paternal genetic role in the development of IUGR.

## Figures and Tables

**Table 1:** Mothers’and fathers’ characteristics by groups.

Parents Characteristics	IUGR	AGA	Stat. (*p*-Value)
	n = 40	n = 21	
Mothers			
Birth weight, g ^a^	2800 (2500 to 3050)	3100 (2900 to 3300)	−2.36 (0.0183)
BMI, kg/m^2^ before pregnancy ^a^	22.5 (20 to 26)	24 (20 to 27)	−0.51 (0.6111)
Gestation ^b^			0.17 (0.9187)
1	17 (42.5)	10 (47.6)	
2	14 (35.0)	7 (33.3)	
>2	9 (22.5)	4 (19.0)	
Parity ^c^			n.a. (0.1349)
1	24 (60.0)	17 (81.0)	
2	10 (25.0)	4 (19.0)	
>2	6 (15.0)	0 (0.0)	
Fathers			
Age, years ^a^	34 (28 to 36)	31 (28 to 36)	0.37 (0.7100)
Ethnicity ^b^			1.36 (0.2436)
Romanian	29 (72.5)	18 (85.7)	
Hungarian	6 (15.0)	2 (9.5)	
Rrom	5 (12.5)	1 (4.8)	
Birth weight, g ^a^	3000 (2800 to 3000)	3400 (3000 to 3500)	−3.61 (0.0003)
BMI, kg/m^2 a^	27.9 (25.5 to 29.5)	26.0 (26.0 to 28.0)	1.37 (0.1695)
BMI class ^c^			
under healthy weight	2 (5.0)	0 (0.0)	n.a. (0.2974)
healthy weight	10 (25.0)	3 (14.3)	n.a. (0.3323)
overweight	19 (47.5)	18 (85.7)	n.a. (0.0037)
obesity grade I	9 (22.5)	0 (0.0)	n.a. (0.0185)
Smoking, yes ^b^	15 (37.5)	7 (33.3)	0.10 (0.7475)
Alcohol, yes ^b^	35 (12.5)	3 (14.3)	0.04 (0.8444)

^a^ data are reported as median (Q1 to Q3), where Q1 is the first quartile and Q3 is the this quartile, comparisons between groups made with Mann-Whitney test; ^b^ data are reported as no. (%), comparisons are made with Chi-squared test or Fisher exact test; ^c^ data are reported as no. (%), comparisons are made with Z-test for proportions. * Romanian vs. others. IUGR—intrauterine growth restriction, AGA—appropriate for gestational age, BMI—body mass index.

**Table 2:** Genetic protective IGF2 profile in the IUGR group.

Characteristic	AA Genotype (n = 5)	AG or GG Genotype (n = 35)	*p*-Value *
Mother age, years	24 (23 to 26)	32 (27.5 to 35)	2.94 (0.0032)
Father age, years	25 (23 to 28)	34 (29 to 37)	2.64 (0.0083)

The values are median and interquartile range (the first quartile to the third quartile). * Mann–Whitney Z-statistic.

**Table 3:** Univariate and multivariate logistic regression analysis.

Independent Variable	IUGR vs. AGA—Univariable		IUGR vs. AGA—Multivariable	
	b [95% CI]	*p*-Value	b [95% CI]	*p*-Value
IGF2, pg/mL	1.000 [0.999 to 1.000]	0.462		
IGF2 genetic profile *	2.187 [0.554 to 8.639]	0.264	0.342 [0.074 to 1.592]	0.172
Mother birth weight, g	0.998 [0.997 to 0.999]	0.022	0.999 [0.997 to 1.001]	0.203
Father birth weight, g	0.998 [0.996 to 0.999]	0.006	0.998 [0.996 to 1.000]	0.032
Father age, years	1.009 [0.913 to 1.115]	0.861		
Mother age, years	1.029 [0.933 to 1.134]	0.573		

IUGR—intrauterine growth restriction; AGA—appropriate for gestational age; BMI—body mass index; b= partial regression coefficients; 95% CI = 95% confidence bounds; * AA = reference. Multivariable model performance: overall model significance: *p*-value = 0.002. Adjusted multiple correlation coefficient: R^2^= 0.293; overall percentage correct classification: 72.1%; Hosmer and Lemeshow Test: *p*-value = 0.210.

## Data Availability

The datasets used and/or analyzed during the current study are available from the corresponding author on reasonable request.
